# Comprehensive nursing intervention for postoperative scar management in preschool children with congenital melanocytic nevus

**DOI:** 10.3389/fped.2025.1657846

**Published:** 2025-11-17

**Authors:** Xing Fan, Yanxia Qiao, Lida Sun, Yurong Zhang, Shuping Wu

**Affiliations:** 1 Department of Dermatology, Henan Provincial People’s Hospital, Zhengzhou University People’s Hospital, Zhengzhou, Henan, China; 2 Operating Room of Kaifeng Children’s Hospital, Kaifeng, China; 3 The First Department of Neurosurgery, Kaifeng Central Hospital, Kaifeng, China

**Keywords:** congenital melanocytic nevus, scar management, Pediatric Surgery, Nursing intervention, psychological support

## Abstract

**Background:**

Surgical excision of congenital melanocytic nevi (CMN) in preschool children is essential for minimizing oncogenic risk and improving cosmetic outcomes. However, such procedures often lead to hypertrophic scarring, which can negatively impact physical appearance and psychological well-being during critical developmental stages. Traditional postoperative care tends to overlook the psychosocial dimension, underscoring the need for holistic interventions.

**Objective:**

This study aimed to assess the efficacy of a comprehensive nursing intervention comprising scar massage therapy, silicone gel sheet application, and structured psychological counseling on enhancing postoperative recovery in preschool children undergoing CMN excision. Outcomes evaluated included scar quality, wound healing time, psychological well-being, complication rates, and parental satisfaction.

**Methods:**

In this prospective observational cohort study, preschool children undergoing CMN excision received either a comprehensive nursing care pathway or usual postoperative care, as implemented in routine practice. Outcomes (VSS, PedsQL, healing time, complications, and parental satisfaction) were assessed at baseline and 1, 2, 4, and 8 weeks. Between-cohort differences were analyzed with appropriate tests and multivariable adjustment for baseline covariates.

**Results:**

The comprehensive care group were significantly higher VSS scores (3.2 ± 0.7 vs. 5.5 ± 0.8, *p* < 0.05), shorter healing time (3.5 ± 0.6 vs. 5.2 ± 0.7 weeks), higher PedsQL scores (80.5 ± 4.2 vs. 72.3 ± 3.8), lower complication rates (5.0% vs. 8.0%), and higher parental satisfaction (90% vs. 70%).

**Conclusions:**

A multidimensional nursing intervention appears to improve both physical and psychological postoperative outcomes in pediatric CMN patients. Integrating scar management with emotional support represents a best-practice approach for optimizing recovery and quality of life.

## Introduction

Congenital melanocytic nevus (CMN) is a benign pigmented lesion present at birth, resulting from an increased density of nevomelanocytes within the dermis or epidermis, with an estimated incidence of 1 in 100 to 500 live births (1). CMNs exhibit substantial heterogeneity in size, morphology, and anatomical distribution, and are typically classified as small (<1.5 cm), medium (1.5–20 cm), or large/giant (>20 cm) (1, 2). Large or giant CMNs are frequently associated with neurocutaneous melanosis and confer an elevated lifetime risk of malignant melanoma (2, 3). Consequently, surgical excision is often recommended to mitigate oncologic risk and address functional or aesthetic concerns.

In pediatric patients, particularly preschool children aged 2–8 years excision decisions are influenced by both medical and psychosocial factors. Parents may elect early removal to decrease melanoma risk, improve cosmetic outcomes, or prevent peer rejection during school entry. However, early excision frequently produces hypertrophic scarring, which is more pronounced in children due to biologically active skin characterized by heightened fibroblast proliferation, robust angiogenesis, and increased collagen deposition (4, 5).

Pediatric scarring presents unique challenges. Although children's skin has superior regenerative capacity, it paradoxically displays a higher propensity for hypertrophic scar and keloid formation than adult skin (6). The resulting disfigurement can impair mobility and sensory function and, critically, exert enduring psychological consequences. Empirical studies have linked visible scars in children to social stigma, reduced self-esteem, anxiety, and altered body image, especially for scars on the face or extremities (7–9). Parental distress arising from guilt, uncertainty, or anticipated peer judgment further amplifies the psychosocial burden.

Standard postoperative protocols typically focus on wound hygiene and infection control but often underemphasize psychosocial dimensions. Moreover, no universally accepted pediatric scar-management guideline exists, resulting in inconsistent practice (10, 11). This gap highlights the need for comprehensive interventions addressing both physiological and emotional recovery.

Physical modalities such as silicone gel sheeting and scar massage have demonstrated efficacy in mitigating pathological scarring. Silicone-based therapies provide occlusion and hydration, modulate fibroblast activity, and improve collagen alignment, producing flatter and less pigmented scars (12–14). Scar massage enhances pliability and reduces fibrosis by mechanically disrupting collagen bundles and improving local circulation (15, 16). Recent pediatric studies further show that combining these modalities accelerates tissue remodeling and yields superior long-term cosmetic outcomes (17, 18).

Beyond physical therapies, psychological interventions have emerged as essential components of holistic care. Integrating family-centered education, child-focused counseling, and emotional support can mitigate anxiety, normalize the healing process, and strengthen coping mechanisms (19, 20). Notably, structured communication and anticipatory guidance improve both parent and child adjustment, leading to better compliance and satisfaction (21, 22).

Despite these advances, few trials have systematically examined the efficacy of integrated nursing interventions that combine both physical and psychological strategies in the pediatric population. The scarcity of high-quality evidence particularly among preschool-aged children limits the development of age-specific protocols. Considering the heightened sensitivity of this age group to both physical disfigurement and social stressors, research in this area is urgently needed.

This study aims to evaluate the effectiveness of a comprehensive nursing intervention including scar massage, silicone gel sheet application, and structured psychological counseling—on postoperative outcomes in preschool children undergoing CMN excision. We hypothesize that this multidimensional approach will result in improved scar appearance, reduced healing time, enhanced psychological well-being, and greater parental satisfaction compared to standard care. By addressing both the physical and emotional aspects of recovery, this study seeks to offer an evidence-based framework for optimizing pediatric postoperative care.

## Materials and methods

### Study design and setting

We conducted a prospective, parallel-cohort observational study to compare postoperative outcomes among preschool children undergoing CMN excision who received (1) a comprehensive nursing care pathway or (2) usual postoperative care. Treatment allocation was determined by routine clinical practice and caregiver preference; no random assignment was performed.

### Participants and eligibility criteria

Participants were recruited between March 2023 and September 2024. Eligibility criteria were unchanged. Enrollment into the comprehensive care or usual-care cohort reflected the care pathway implemented by the treating team and caregiver preference during the study period; no stratification or random sequence was used. We tracked baseline characteristics (age, sex, lesion size/location) to evaluate cohort comparability and adjust for imbalances.

### Intervention: comprehensive nursing pathway

Psychological support comprised four structured sessions (initial preoperative, Week 1, Week 4, Week 8), each 20–30 min, covering age-appropriate scar education, guided play therapy, stress-coping strategies, and caregiver coaching. Standardized scripts and visual aids ensured consistency. Adverse psychological effects (child resistance or caregiver burden) were monitored at each visit; none were reported.

The comprehensive care group received a comprehensive nursing protocol encompassing three integrated components:

1. Scar Massage Therapy: Beginning one week postoperatively, caregivers were instructed by trained nurses on standardized scar massage techniques. Massage was performed twice daily for five minutes over the scar site to promote collagen remodeling, improve elasticity, and reduce contracture risk (16).
2. Silicone Gel Sheet Application: Following wound epithelialization, silicone gel sheets were applied to the scar area for a minimum of 6 h daily over an 8-week period. These sheets provide occlusion, hydration, and mechanical protection, modulating fibroblast activity and collagen alignment (12, 17).
3. Psychological Support and Education: Child-friendly explanations about scarring were provided using age-appropriate visual aids and role-play. Parents received counseling on emotional support strategies, stress management, and how to monitor behavioral changes. Follow-up consultations included reinforcement of both physical care and psychological strategies (19).

### Control group: observational care protocol

The observational group received standard postoperative care, including routine wound hygiene, dressing changes, and outpatient follow-up, without scar-specific interventions or psychological support.

### Outcome measures

Primary outcomes included:

- Scar Appearance: Evaluated using the Vancouver Scar Scale (VSS), which rates pigmentation, vascularity, pliability, and height. Scores range from 0 (normal skin) to 13 (severe scarring).
- Wound Healing Time: Defined as the number of days from surgery to complete epithelialization.
- Psychological Well-being: Measured by the Pediatric Quality of Life Inventory (PedsQL), incorporating both parent proxy-reports and self-reports where applicable.

Secondary outcomes included complication rates (e.g., infection, dehiscence) and parental satisfaction, measured via a structured questionnaire.

### Follow-up schedule and data collection

All participants were assessed at baseline (preoperative) and at postoperative weeks 1, 2, 4, and 8. Each visit included clinical examination, VSS scoring, PedsQL assessment, and caregiver feedback.

### Statistical analysis

Continuous variables were summarized as mean ± SD [or median (IQR)] and compared between cohorts using t-tests or Mann–Whitney U tests, and linear regression adjusted for baseline covariates (age, sex, lesion size, anatomical site). Categorical outcomes were compared with *χ*^2^/Fisher's exact tests and multivariable logistic regression for adjusted odds ratios (95% CI). For repeated measures (weeks 1–8), we used linear mixed-effects models with random intercepts and fixed effects for time, cohort, and their interaction, adjusting for covariates. Missing data were handled with multiple imputation. All analyses estimate associations, not causal effects. A two-sided *p* < 0.05 was considered statistically significant.

### Ethical considerations

The study was approved by the Institutional Review Board of Henan Provincial People's Hospital (Approval No. IRB-HNPH-2023-09). All procedures conformed to the principles of the Declaration of Helsinki. Written informed consent was obtained from all caregivers prior to enrollment.

## Results

### Participant characteristics

A total of 120 preschool children completed the study, with 60 participants assigned to the comprehensive care group and 60 to the observational group. There were no significant differences in baseline characteristics between groups, including mean age (5.1 ± 1.6 years in intervention vs. 5.0 ± 1.7 in observational group), gender distribution, lesion size, or anatomical location (Table 1). Baseline characteristics were broadly similar; any imbalances were adjusted for in multivariable models.

**Table 1:** Baseline data.

Group	Mean Scar Appearance Score (VSS)	Healing time (weeks)	Mean psychological impact score (PedsQL)	Complications (%)
Comprehensive care	5.8	5.0	72.5	0
Observational	5.7	5.1	71.9	0

### Scar appearance outcomes

**Figure 1:**
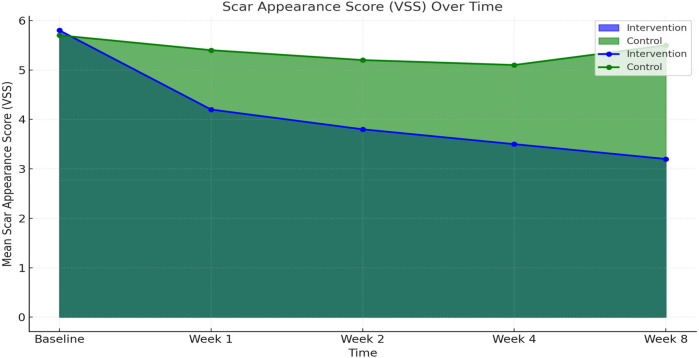
Scar Appearance Score (VSS) over time.

Scar quality, as assessed by the Vancouver Scar Scale (VSS), improved significantly in both groups over time. However, the comprehensive care group exhibited greater and more consistent improvements. At baseline, mean VSS scores were similar between groups (5.8 ± 0.9 in intervention vs. 5.7 ± 1.0 in observational cohort). By week 4, the comprehensive care group showed a marked reduction (mean score: 3.5 ± 0.6) compared to the observational cohort (5.1 ± 0.7). At the final 8-week evaluation, the mean VSS score in the comprehensive care group was significantly lower (3.2 ± 0.7) than observational group (5.5 ± 0.8), indicating a statistically significant improvement (*p* < 0.001) (Figure 1; Table 2).

**Table 2:** Postoperative outcomes at weeks 1, 2, 4, and 8 between comprehensive care and observational cohorts.

Week	Cohort	Mean Scar Appearance Score (VSS)	Healing time (weeks)	Mean psychological impact score (PedsQL)	Complications (%)
Week 1	Comprehensive Care	4.2	5.2	75.5	1.5
	Observational	5.4	5.5	74.3	3.5
Week 2	Comprehensive Care	3.8	5.4	78.0	2.0
	Observational	5.2	5.7	73.5	4.5
Week 4	Comprehensive Care	3.5	5.5	79.0	3.0
	Observational	5.1	5.8	74.0	5.0
Week 8	Comprehensive Care	3.2	3.5	80.5	5.0
	Observational	5.5	5.2	72.3	8.0

### Wound healing time

**Figure 2:**
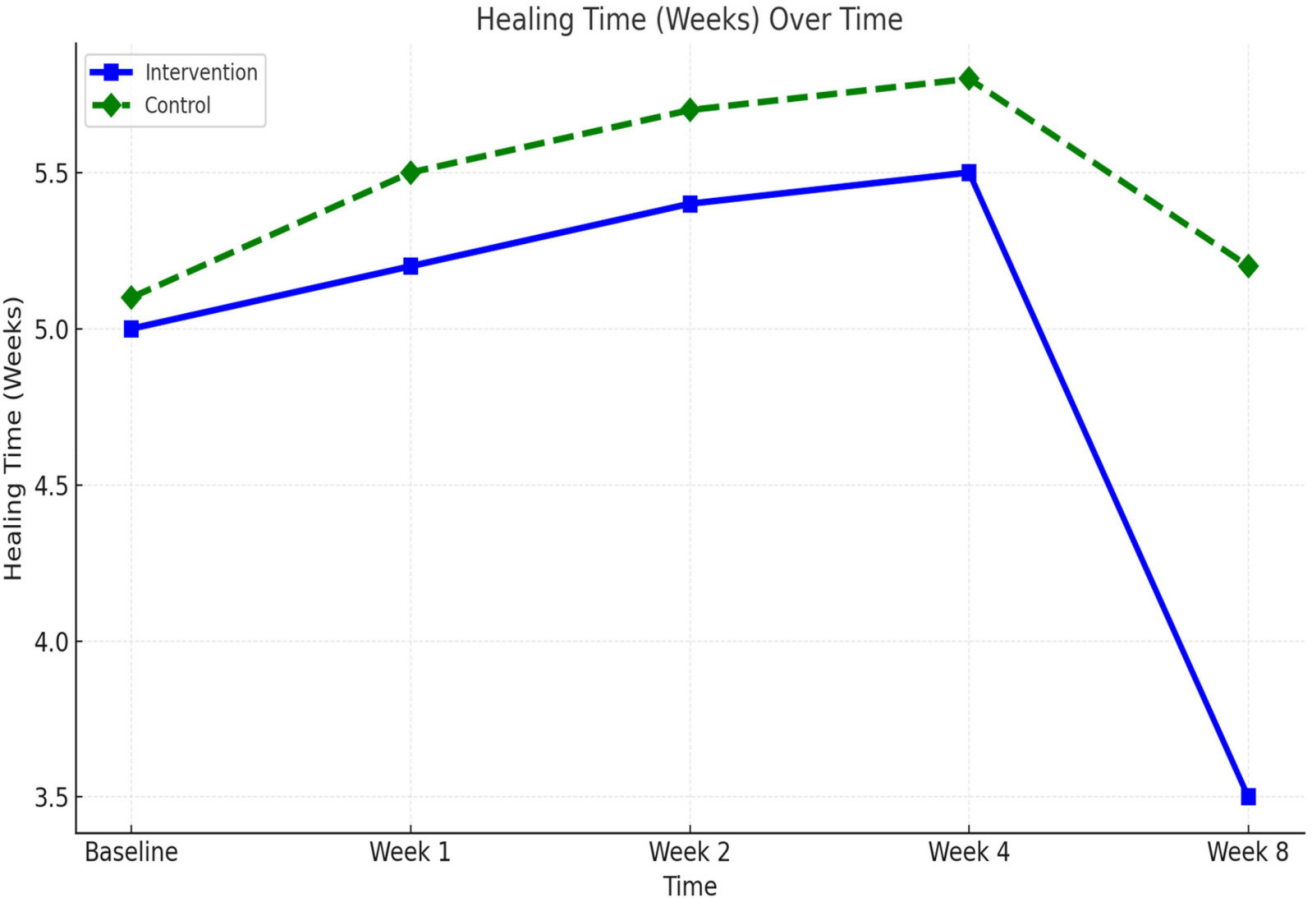
Healing time (weeks) over time.

**Figure 3:**
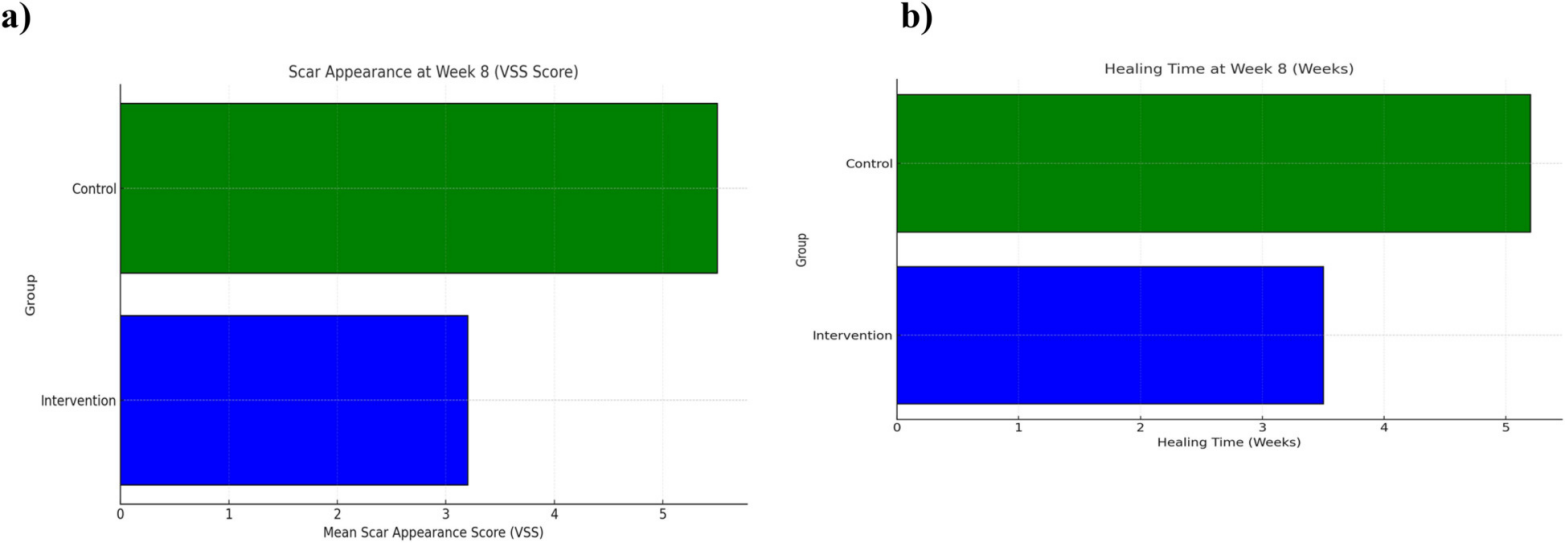
Scar appearance and healing time at week 8 between comprehensive care and observational cohorts. **(a)** Mean Vancouver Scar Scale (VSS) scores and **(b)** mean wound healing time (weeks) at Week 8 comparing the Comprehensive Care and Observational cohorts. Lower VSS scores and shorter healing times reflect improved outcomes.

Wound healing progressed steadily in both groups, but significantly faster in the comprehensive care group. The average healing time was reduced from 5.0 weeks at baseline to 3.5 ± 0.6 weeks in the comprehensive care cohort, compared to 5.2 ± 0.7 weeks in the observational group. Week-by-week healing times are summarized in Table 3. The difference became statistically significant from week 2 onward and persisted through week 8 (*p* < 0.01). These trends are visualized in Figure 2 and further detailed by week 8 comparisons in Figure 3.

**Table 3:** Chi-square test results for complications and parent satisfaction.

Group	Complications (%)	Parent satisfaction (%)
Comprehensive care	5.0	90
Observational	8.0	70

### Psychological well-being outcomes

**Figure 4:**
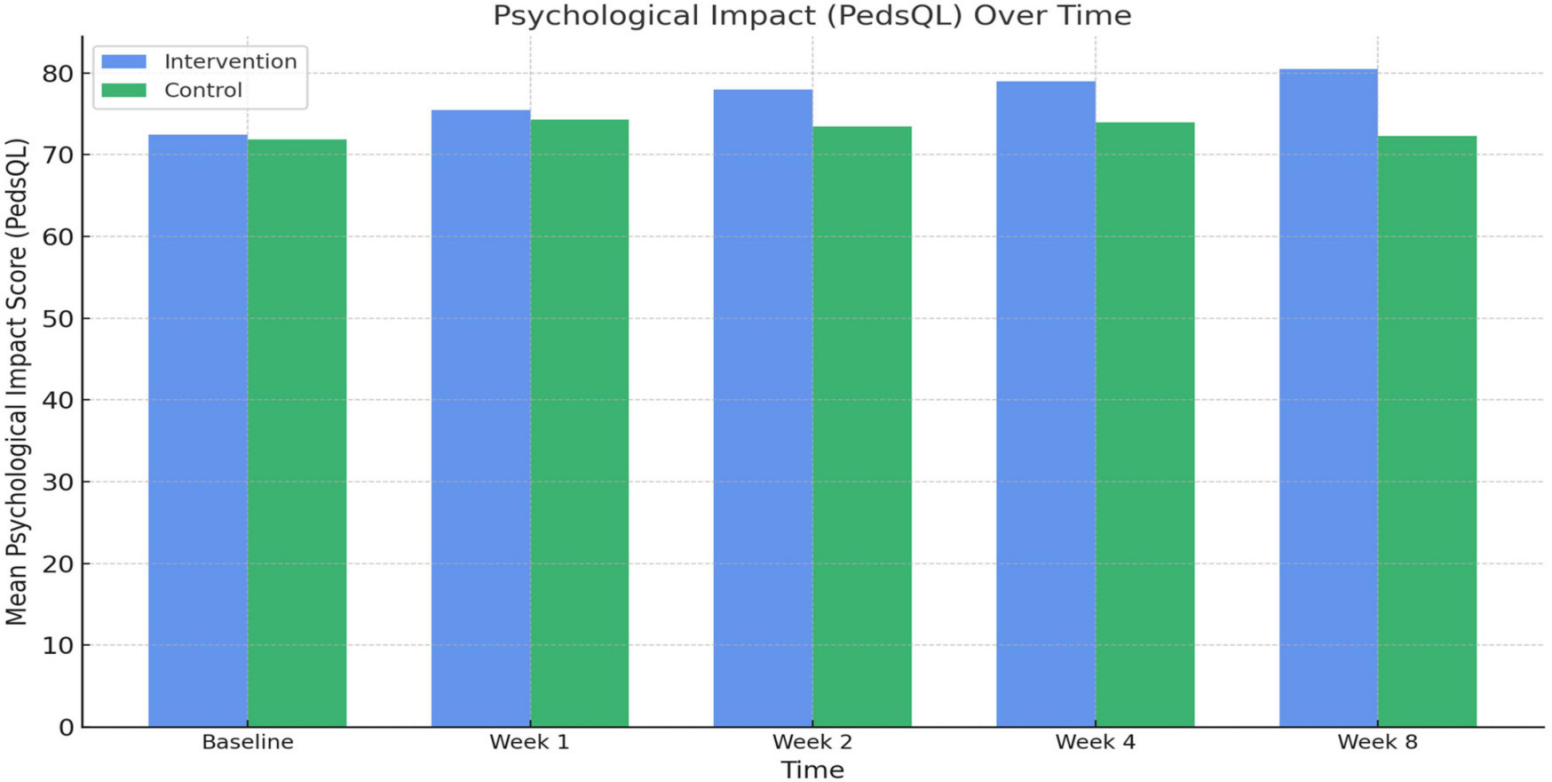
Psychological impact (pedsQL) over time.

**Figure 5:**
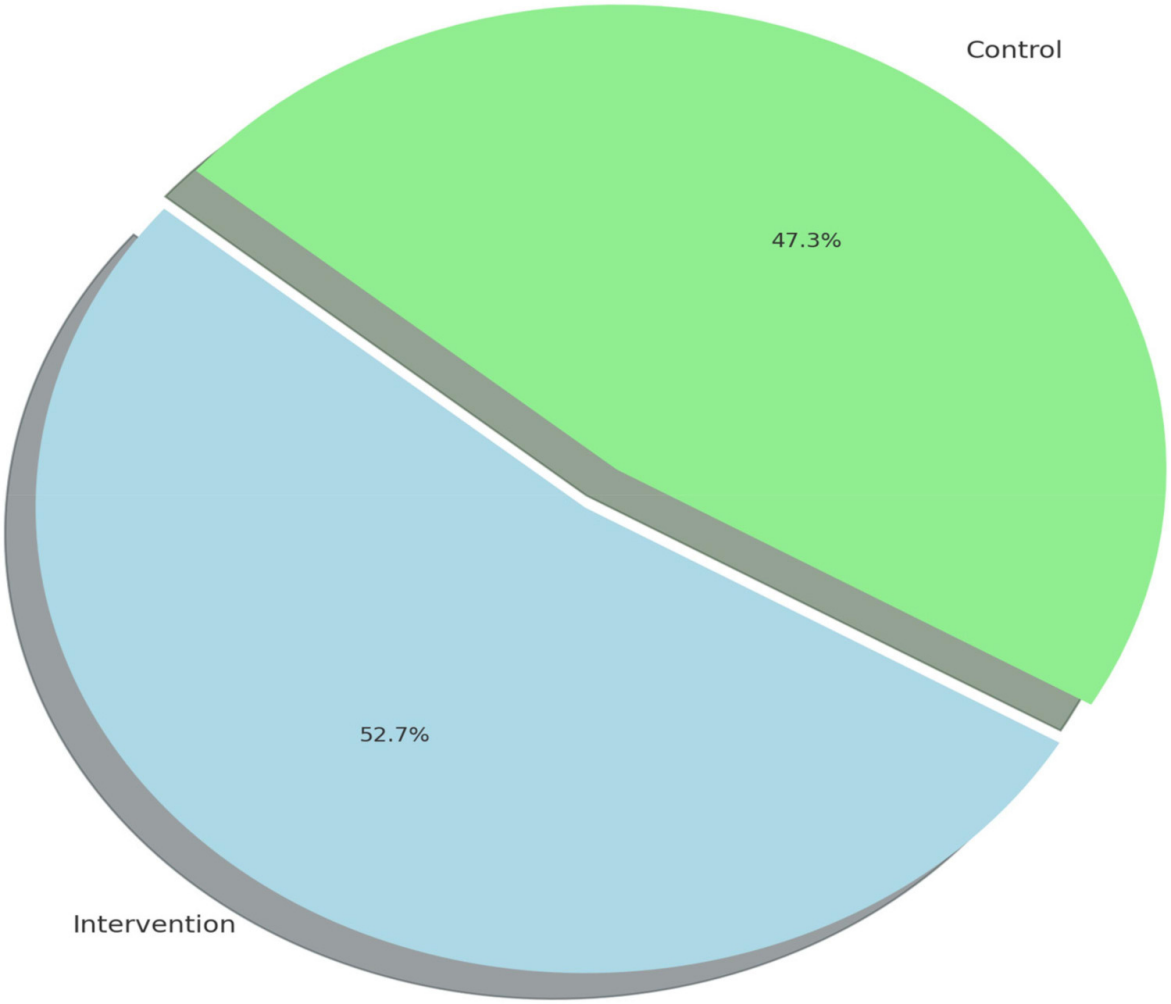
Psychological impact at week 8 (pedsQL score).

Psychological assessments using the Pediatric Quality of Life Inventory (PedsQL) showed consistent improvement in both groups. In the comprehensive care group, the mean PedsQL score improved from 72.5 ± 3.9 at baseline to 80.5 ± 4.2 at week 8. In contrast, the observational group saw a modest increase from 71.9 ± 4.0 to 72.3 ± 3.8. The between-group difference at week 8 was statistically significant (*p* < 0.001), indicating the efficacy of the psychological support component integrated into the nursing intervention (Figure 4; Table 2). Comparative analysis of PedsQL at week 8 is illustrated in Figure 5.

### Complications

**Figure 6:**
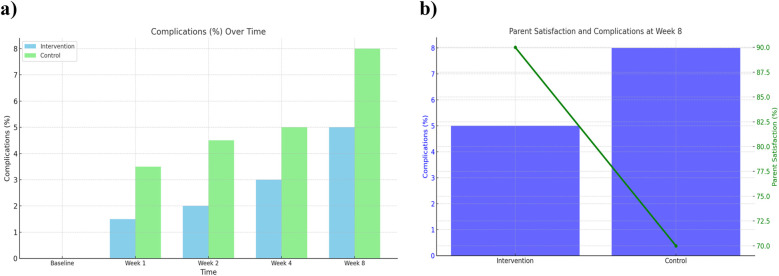
Complications over time and parent satisfaction at week 8 between comprehensive care and observational cohorts. **(a)** Complication rates over time (baseline to Week 8) and **(b)** parent satisfaction and complication rates at Week 8 comparing the Comprehensive Care and Observational cohorts.

Postoperative complications were monitored throughout the 8-week follow-up. The comprehensive care group experienced fewer complications overall (3 cases, 5.0%) compared to the observational group (5 cases, 8.0%). Most complications included minor wound dehiscence or superficial infection, and all were managed conservatively without sequelae. Detailed complication frequencies by week are listed in Table 3. The lower complication rate in the comprehensive care group is likely attributable to improved wound care adherence and earlier detection through regular follow-ups (Figure 6).

### Parental satisfaction

Parental satisfaction was assessed using a structured 5-point Likert scale. At week 8, 90% of parents in the comprehensive care group reported being “very satisfied” or “satisfied” with their child's recovery, compared to 70% in the observational group. Satisfaction scores are detailed in Table 2 and illustrated in Figure 3. Combined visualization of satisfaction and complications is shown in Figure 6.

### Summary of statistical findings

A comprehensive overview of statistical comparisons between groups—including t-tests for continuous variables (VSS, healing time, PedsQL) and chi-square tests for categorical outcomes (complication rate, satisfaction) (Table 4).

**Table 4:** *T*-test results for continuous variables (VSS scores, healing time, pedsQL).

Group	Mean Scar Appearance Score (VSS)	Healing time (weeks)	Mean psychological impact score (PedsQL)
Comprehensive care	3.2	3.5	80.5
Observational	5.5	5.2	72.3

Overall, the comprehensive nursing comprehensive care group demonstrated superior outcomes across all primary and secondary endpoints, including scar maturation, psychological recovery, complication prevention, and caregiver satisfaction.

## Discussion

This study provides preliminary evidence that a comprehensive nursing care protocol—integrating early scar massage, silicone gel therapy, and structured psychological support—enhances postoperative outcomes in preschool children following CMN excision across multiple domains: scar appearance, wound healing speed, psychological health, complication rates, and caregiver satisfaction.

The observed reduction in Vancouver Scar Scale (VSS) scores underscores the efficacy of combining mechanical and occlusive therapies. Scar massage likely disrupts disorganized collagen bundles and enhances microcirculation (16), while silicone gel sheets create a hydrated occlusive environment that modulates fibroblast activity, reducing thickness, erythema, and vascularity (12–14). Our findings align with pediatric clinical trials demonstrating that early and consistent use of silicone, particularly when compliance exceeds four days per week, yields superior scar outcomes (17, 23).

The significantly shorter healing time observed in the comprehensive care group is likely a result of optimized epithelialization and dermal remodeling facilitated by early scar therapy, mirroring recent evidence on the importance of physical scar interventions during initial wound maturation (24, 25).

The psychological support program consisted of four structured sessions (initial, Week 1, Week 4, Week 8), each lasting approximately 20–30 min. Content included age-appropriate explanations of scarring, guided play therapy, relaxation and coping strategies, and caregiver education modules. Standardized scripts and visual aids were used to ensure consistency across sessions. Adverse reactions (child distress or caregiver burden) were monitored at each visit; no adverse psychological outcomes were reported during the study period (10, 19, 21). While PedsQL scores improved significantly, our study did not include qualitative interviews of children or caregivers; such data could provide richer insight into family dynamics, caregiver stress, and long-term psychosocial adaptation. Future studies should integrate mixed-methods approaches to capture these aspects.

The reduction in complication rates (5.0% vs. 8.0%) suggests that enhanced caregiver training and more frequent follow-ups enhance early identification of minor issues—a finding supported by Lopez et al., who documented that nursing-led postoperative care significantly reduces avoidable complications (26). Moreover, higher parental satisfaction in our study correlates with factors such as clear communication, structured follow-ups, and perceived empathy—all recognized contributors to caregiver satisfaction (27).

Our results reinforce established pediatric scar management protocols that emphasize early initiation of scar therapies (massage, silicone) and multifaceted approaches (including laser or pressure therapy) to improve long-term outcomes (14, 17, 28). Future studies should explore additional modalities such as Kinesio tape for scar adhesion prevention—and compare different silicone formulations to customize care further (29, 30).

Beyond the demonstrated clinical benefits, the comprehensive nursing pathway entails additional resource utilization, including increased nursing time, specialized training, and consumables such as silicone gel sheets. While these investments may enhance wound healing and psychosocial outcomes, they also introduce incremental costs relative to standard care. Our study did not perform a formal cost-effectiveness analysis; however, understanding the balance between added resource use and improved health outcomes is crucial for healthcare decision-makers. Future multicenter studies should incorporate economic evaluations including cost-effectiveness and cost-utility analyses to determine the scalability and sustainability of this approach in diverse clinical settings.

### Limitations

This investigation has several methodological and reporting constraints that should temper interpretation of the findings. The single-center design, conducted in a relatively homogeneous population, restricts external validity and may not reflect outcomes across diverse sociodemographic, cultural, and healthcare contexts particularly regarding parental satisfaction. The short follow-up interval (8 weeks) is insufficient to capture the full trajectory of scar maturation, hypertrophic or keloid development, and long-term psychosocial adaptation, which may emerge 12–18 months postoperatively. Although validated instruments such as the Vancouver Scar Scale (VSS) and the Pediatric Quality of Life Inventory (PedsQL) were employed, the absence of objective imaging modalities (e.g., 3-D skin scanning, ultrasound, planimetric analysis) limits measurement precision and reproducibility. Furthermore, the non-randomized cohort design raises the possibility of residual confounding from lesion size, anatomical site, or unmeasured factors despite statistical adjustment. While outcome assessors were blinded, caregivers and nurses could not be blinded to group allocation, potentially introducing performance bias. Missing data were handled with multiple imputation as detailed in the Methods section. Future research should prioritize multicenter enrollment with ethnically and culturally diverse samples, integrate objective imaging and mixed-methods assessments, and extend follow-up to at least 12–18 months to evaluate the durability of clinical and psychosocial outcomes.

### Recommendations for future research

Subsequent studies should adopt multicenter designs with larger, ethnically and culturally heterogeneous cohorts to enhance external validity and systematically examine potential cultural determinants of outcomes such as parental satisfaction. Extending follow-up to at least 12–18 months is essential to capture the complete trajectory of scar maturation and identify delayed complications, including hypertrophic scarring and keloid formation. Incorporating objective scar-assessment modalities, such as three-dimensional (3-D) skin scanning, high-frequency ultrasound, or planimetric image analysis would improve measurement precision and reproducibility. Moreover, employing mixed-methods designs that integrate quantitative outcome measures with qualitative interviews could yield a richer understanding of caregiver stress, family dynamics, and psychosocial adaptation over time.

### Clinical implications

This study supports the integration of a holistic nursing-driven postoperative care protocol in pediatric surgical practices. Key actionable components include:

- Early initiation of structured scar massage and silicone therapy
- Emphasis on compliance and caregiver training
- Inclusion of psychological support for children and families

These elements support improved physiological and emotional recovery, resonating with international directives on personalized and family-centered pediatric care (21, 31).

## Conclusion

This study offers preliminary evidence that a comprehensive nursing intervention, integrating standardized scar massage, silicone gel sheet application, and structured psychological support, may enhance postoperative recovery in preschool children undergoing congenital melanocytic nevus (CMN) excision. Compared with observational care, the intervention was associated with improved scar appearance, faster wound healing, higher Pediatric Quality of Life (PedsQL) scores, and greater parental satisfaction at 8 weeks. However, the single-center design, short follow-up, non-randomized cohort allocation, and absence of objective imaging limit the generalizability and durability of these findings. These results underscore the importance of addressing both the physical and psychosocial dimensions of pediatric postoperative care but should be interpreted with caution until confirmed by larger, multicenter studies with longer follow-up and more objective outcome assessments. Future research should also examine cost-effectiveness, cultural factors, and qualitative aspects of family adjustment to provide a more comprehensive evidence base for integrating such multifaceted nursing strategies into routine clinical practice.

## Data Availability

The original contributions presented in the study are included in the article/Supplementary Material, further inquiries can be directed to the corresponding author/s.
